# Sympathetic Nerve-Mediated Fellow Eye Pain During Sequential Cataract Surgery by Regulating Granulocyte Colony Stimulating Factor CSF3

**DOI:** 10.3389/fncel.2022.841733

**Published:** 2022-02-24

**Authors:** Zheng Fan, Caixia Fan, Benxiang Qi, Bin Zhang, Wenfeng Li, Xia Qi, Xiaomin Liu, Bi Ning Zhang, Yusen Huang

**Affiliations:** ^1^Medical College of Qingdao University, Qingdao, China; ^2^State Key Laboratory Cultivation Base, Shandong Provincial Key Laboratory of Ophthalmology, Eye Institute of Shandong First Medical University, Qingdao, China; ^3^Qingdao Eye Hospital of Shandong First Medical University, Qingdao, China; ^4^Department of Medical Oncology, The Affiliated Hospital of Qingdao University, Qingdao, China

**Keywords:** contralateral eye, CSF3, cytokine, pain, sympathetic nerve

## Abstract

Patients were found to experience more pain during their second eye cataract surgery compared with their first eye surgery. This study aimed to explore the inflammatory alterations along time in the fellow eye after the first eye surgery and to reveal the underlying mechanism. Eighty patients with bilateral cataracts were recruited and were divided into four groups based on the time of having the second eye surgery. The second eye aqueous humor samples were collected just before surgery and analyzed by mass spectrometry and PCR array. Cytokine activity was enriched in the aqueous humor of the contralateral eye with granulocyte colony-stimulating factor CSF3 significantly upregulated at both gene and protein levels. Rabbits with or without superior cervical ganglionectomy (SCGx) were subjected to lensectomy to mimic human situations. In both human and rabbit models, the fellow eye CSF3 peaked at 1 week post the first eye surgery. Consistently, more neutrophils were recruited to the contralateral eye aqueous humor. Corneal sensitivity and trigeminal electrophysiology were recorded to imply the pain severity in rats receiving capsulorrhexis with or without SCGx. A more intense pulse was detected in the contralateral trigeminal ganglion after the rat received one eye surgery. SCGx could effectively reduce the fellow corneal sensitivity and trigeminal nerve pain. These alterations were under direct regulation of the sympathetic nerves on the surgical eye side. Our results suggest that CSF3 and sympathetic activity could serve as potential analgesic targets during ocular surgeries.

## Introduction

Cataract surgery is the most common therapeutic modality for patients with cataracts. Recent innovations on foldable intraocular lenses (IOL) and instruments as well as small or micro-incision phacoemulsification techniques bring less risk and side effects to patients. Staged cataract surgical operation for bilateral eyes is more commonly performed than simultaneous cataract surgery in both eyes, mainly to avoid the catastrophic bilateral endophthalmitis (Kushner, 2010). However, during clinical practice, there are still patients complaining of eye pain, dry eyes, foreign body sensation, or other discomfort symptoms. Intriguingly, these symptoms were more intense during the surgery in the second eye than that in the first eye, especially for pain perception, which had been noticed extensively (Tan et al., 2011; Ursea et al., 2011; Adatia et al., 2015; Jiang et al., 2015; Yu et al., 2016; Liu et al., 2020). Psychological factors like anxiety and perception during the first surgery would influence the patient’s eye sensation (Nijkamp et al., 2002; Ang et al., 2007; Adatia et al., 2015) while physiological factors, such as repeated penetrating injuries, might have triggered a sub-clinical sympathetic inflammatory reaction in the fellow eye (Zhu et al., 2015).

Cytokines are secretory proteins that mediate and control immune and inflammatory responses. Pain is closely related to inflammation, and the latter could cause spontaneous hypersensitivity to pain by directly activating the corresponding receptors in the sensory nerve endings for pain perception (Schweizerhof et al., 2009). The comparison of the expression level of aqueous humor inflammatory cytokines during the first-eye or second-eye cataract surgery illustrates an alteration of cytokine profiles in these two eyes (Zhu et al., 2015), indicating that the inflammatory status in the contralateral eye might be affected by the surgical process in the first eye. However, the dynamic changes of cytokines in the aqueous humor of the contralateral eye have not been reported, and the underlying mechanisms have not been explored yet. Numerous studies have been conducted to reveal the link between sympathetic nerves, its neurotransmitter norepinephrine, and immune cell activities (Sharma and Farrar, 2020), but which immune cell plays a role during eye pain and whether it is regulated by sympathetic nerves are still unknown. Therefore, we explored the role of sympathetic nerves in the modulation of eye hypersensitivity as well.

The aim of the present study was to identify the initial inflammatory response in the anterior chamber of the contralateral eye following cataract surgery in the first eye and to explore the regulatory mechanisms and the efferent cells during the fellow eye hyperalgesia.

## Materials and Methods

### Ethics and Data Availability

Patient recruitment and the study design were following the tenets of the Declaration of Helsinki and were approved by the Ethics Committee of Qingdao Eye Hospital, with informed consent obtained. The study was registered on the Chinese Clinical Trial Registry (ChiCTR2100050077). Data generated from mass spectrometry and PCR array have been uploaded to OMIX^1^ with the accession number OMIX690. Ethics approval for animal studies was obtained from the Animal Investigation Committee of Eye Institute of Shandong First Medical University. All animal procedures were performed in accordance with the Association for Research in Vision and Ophthalmology (ARVO) statement.

### Pain Score Questionnaire

Patients who received phacoemulsification cataract surgery at Qingdao Eye Hospital were randomly recruited for the pain survey (Jiang et al., 2015; Yu et al., 2016). All surgeries for these patients were performed under the same conditions by the same surgeon (YSH). Patients with glaucoma, high intraocular pressure, lens dislocation, and posterior capsular rupture were excluded from the survey. The questionnaire was fully explained to every participant. The feelings of the patient after the first and second cataract surgeries were recorded as well.

### Cataract Patient Aqueous Humor Collection

A total of eighty patients undergoing cataract surgeries for both eyes at Qingdao Eye Hospital were recruited, and these patients were further divided into four groups based on the time they are having their contralateral eye cataract surgery, specifically, at 3 days (3D), 1 week (1W), 2 weeks (2W), and 1 month (1M) after their first eye cataract surgery. A total of 20 patients having cataract surgery for the first time were recruited as the control group (Con). The demographic data of the patients are shown in Table 1. All surgeries were conducted by the same surgeon (YSH). Routinely, topical anesthesia using Oxybuprocaine hydrochloride 0.4% eye drops (Santen, Osaka, Japan) was applied at the beginning of the operation. The conjunctive sac was washed with povidone-iodine and an ample amount of normal saline. Aqueous humor samples (150–200 μL) were collected by corneal paracentesis with a 29-gauge insulin syringe (BD Ultra-Fine, Franklin Lakes, NJ, United States) inserted into the anterior chamber, and the sample was immediately stored at −80°C till further use. A 2.8 mm clear corneal incision was made at the 11 o’clock position for all patients. Conventional continuous curvilinear capsulorhexis, hydrodissection, chopping, nucleus rotation, and phacoemulsification were conducted. An optimal IOL was implanted by a dedicated injector afterward. Finally, the incision was hydrated with a balanced salt solution and verified for water tightness after the aspiration of residual sodium hyaluronate.

**TABLE 1:** Patient characteristics.

Demographic data	Mass spectrometry					PCR array				
	First	Second eye				First	Second eye			
		3D	1W	2W	1M		3D	1W	2W	1M
*N*	10	10	10	10	10	10	10	10	10	10
Age_Mean, y	70.2	71.5	71.8	66.9	69.6	69.4	70.8	70.9	69.9	69.1
Age_SD, y	5.35	4.45	6.10	9.74	6.18	5.70	4.50	3.07	4.33	4.62
Male/Female	5/5	4/6	6/4	5/5	4/6	4/6	3/7	3/7	5/5	4/6
The first eye, OD/OS	3/7	6/4	5/5	5/5	6/4	5/5	4/6	3/7	6/4	7/3

### Mass Spectrometry

Aqueous humor mass spectrometry was conducted by Cloud-Seq Biotech (Shanghai, China) with a Q Exactive Orbitrap Mass Spectrometer (Thermo Fisher Scientific). Detailed detection procedure and data analysis were as described in a previous study (Zhang et al., 2021).

### PCR Array

Aqueous humor RNA was isolated with Trizol (Invitrogen, Carlsbad, CA, United States) and purified by an RNeasy MinElute Cleanup Kit (Qiagen, United States). Complementary DNA (cDNA) was synthesized with a SuperScript III Reverse Transcriptase (Invitrogen, Carlsbad, CA, United States). The PCR array for cytokines and chemokines was conducted with an RT^2^ Profiler PCR Array Kit (Qiagen, 330231 PAHS-150ZA, United States) according to the manufacturers’ instructions. A total of 90 cytokines or chemokines were quantified in each sample.

### Rabbit Lensectomy

New Zealand white rabbits around 2 months old were randomly allocated into three groups receiving surgeries at different time points. Both male and female rabbits were used. The rabbits were anesthetized by an intravenous injection of 3% pentobarbital sodium salt solution through the marginal ear vein (30 mg/kg) and had phacoemulsification on the right eyes by the same surgeon as the human subjects (YSH). To be specific, the rabbit had 0.5% Levofloxacin (Cravit; Santen, Osaka, Japan) eye drops three times a day for 2 days. The rabbit had its pupil dilated with tropicamide phenylephrine eye drops (Mydrin-P; Santen, Osaka, Japan) 30 min before the surgery, and had the eye locally anesthetized by oxybuprocaine hydrochloride 0.4% eye drops (Santen, Osaka, Japan) 15 min before surgery. Surgical procedures were conducted under the OPMI-Lumera 700 microscope (Carl Zeiss, Germany). A total of 150–200 μL aqueous humor samples were collected by corneal paracentesis with a 29-gauge insulin syringe inserted into the anterior chamber. Then a 2.8 mm clear corneal incision was made at the 11 o’clock position, with a paracentesis site at the 3 o’clock position. Afterward, an ophthalmic viscoelastic device (Qisheng Biologic Preparation, Shanghai, China) was injected into the anterior chamber. Manual continuous curvilinear capsulorhexis was conducted, followed by phacoemulsification and cortical aspiration with the WhiteStar Signature Phacoemulsification System (Abbott Medical Optics, United States). Incisions were closed by 10–0 sutures (MANI Inc., Tochigi, Japan). Dexamethasone eye drops (TobraDex, Alcon, United States), pranoprofen eye drops (Senju Pharmaceutical, Japan), and tropicamide phenylephrine eye drops (Mydrin-P; Santen, Osaka, Japan) were applied to the rabbit eyes three times a day for 14 days after the surgery.

### CSF3 Expression Validation in Rabbit

Rabbit aqueous humor was collected before phacoemulsification at 3 days (3D), 1 week (1W), 1 month (1M) after surgery by the 29-gauge insulin syringe (BD Ultra-Fine, Franklin Lakes, NJ, United States). Aqueous humor RNA was extracted with a TransZol Up Plus RNA Kit (TransGen Biotech, Beijing, China) and the cDNA was synthesized using the PrimeScript cDNA Synthesis Kit (Takara, Otsu, Japan). Premix Taq (Takara, Otsu, Japan) was used for PCR reaction. The quantitative PCR (qPCR) primers used were: rabbit *CSF3* (XM_017349312.1) 5′-CGACTTTGCCACCACCATCT-3′, 5′-GTCAGCTCCAGGAAGCTCTG-3′; rabbit *GAPDH* (NM_001082253.1) 5′-CGCCTGGAGAAAGCTGCTAA-3′, 5′-CCCCAGCATCGAAGGTAGAG-3′.

The rabbit CSF3 ELISA kit was purchased from Jiangsu Meimian Industrial Co., Ltd. (Yancheng, Jiangsu, China). Rabbit aqueous humor was collected and centrifuged at 3,000 rpm for 15 min to obtain the supernatant. ELISA was performed according to the manufacturer’s instructions.

### Lens Extraction in Rats

Sprague-Dawley rats of 10-week-old were used in this study. Intraperitoneal injection of 3.0% pentobarbital sodium was given to the rats to induce general anesthesia. The pupils were dilated with tropicamide phenylephrine eye drops (Santen, Osaka, Japan). In the extracapsular lens extraction (ECLE) model, a corneal incision was made with a 2.2 mm stab knife, and the anterior chamber was injected with sodium hyaluronate 1.5% (Qisheng Biologic Preparation, Shanghai, China). The anterior capsule was punctured with a discussion needle and the capsulorhexis was conducted with capsulorhexis forceps (MR-F212T-7, Mingren, Suzhou, China). The corneal incision was then extended to approximately 150 degrees with Vannas scissors, followed by hydrodissection and lens removal. Finally, the incision was closed with 10–0 sutures, and topical Tobradex ointment (0.3% tobramycin and 0.1% dexamethasone; Alcon Laboratories) was administered. The ECLE model of rats was only used in the electrophysiological experiment.

### Capsulorhexis in Rats

The surgical procedures were performed in a similar way as the ECLE, with the exception that the corneal incision was not extended after the completion of the capsulorhexis, but was closed with 10–0 sutures instead. Topical Tobradex ointment (0.3% tobramycin and 0.1% dexamethasone; Alcon Laboratories) was administered. Rats undergoing capsulorhexis without lens extraction were used for the flow cytometry and corneal sensitivity tests.

### Superior Cervical Ganglionectomy

Superior cervical ganglionectomy (SCGx) was performed on rabbits and rats with modification of the published protocols (Zareen and Greene, 2009; Savastano et al., 2010). Briefly, an incision was made at the ventral side of the neck to let the superficial cervical fascia and mandibular glands be exposed. The superficial layer of muscles including the sternohyoid muscles, the sternomastoid muscle, the omohyoid muscle, and the digastric muscles were exposed, and the carotid triangle was localized. The carotid sheath was removed, and the common carotid artery was observed. The SCGx was exposed by displacing the external and internal carotid arteries. The SCG was gently pulled until their avulsion. The palpebral ptosis is used as the indicator of successful SCGx.

Either the right or the left side of the two SCGs was removed for each rat. Rats around 2-month-old of either sex were randomly allocated into SCGx groups or the control group. The success of SCGx was determined by the observation of blepharoptosis and pupil constriction 3 weeks after the surgery. SCGx animals that recovered to good conditions were used for further experiments.

### Neutrophil Depletion With Fucoidan

Two-month-old rats of either sex were randomly divided into the fucoidan treatment group and the control group. Fucoidan (f8190, Sigma, United States) was dissolved with saline and was applied through rat tail vein injection using a dose of 25 mg/kg body weight at 15 min after the right eye capsulorhexis in the fucoidan group. Controls only underwent right eye capsulorhexis. The effect of neutrophil depletion after fucoidan application was determined by flow cytometry and corneal sensitivity assessment.

### Flow Cytometry

Rats of either sex with or without SCGx had capsulorhexis in their right eyes (OD) and had the OD aqueous humor samples collected 24 h after surgery. One week later the same rats had capsulorhexis with their left eyes (OS) and had the OS aqueous humor samples collected 24 h after surgery. The freshly collected aqueous humor (about 15 μl) was diluted to 1 ml with a fluorescence activated cell sorting (FACS) buffer [phosphate-buffered saline (PBS) + 2% fetal bovine serum (FBS)]. In total, 100 μl of liquid were collected from each of the diluted aqueous samples and were combined to serve as the negative control (Neg). The only difference between the negative control and the stained samples is whether the primary antibody is added. Samples were centrifuged at 500 g for 5 min at 4°C and resuspended with 100 μl FITC-Ly6G antibody solution (1:100, ab25024, Abcam, United States) and stained for 40 min in the dark at 4°C. After staining, the cells were washed with 1 ml FACS buffer and resuspended with 500 μl FACS buffer. Cells were examined on a CytoFLEX Flow Cytometer (Beckman Coulter). FACS data was quantified with the FlowJo software.

### Whole-Mount Neuron Immunostaining

In brief, rabbit corneas were collected and fixed in Zamboni’s fixative solution for 4 h. After washing with PBS for three times, the corneas were blocked by PBS with 0.3% Triton X-100, 5% donkey serum overnight at 4°C, and subsequently incubated in the same incubation buffer with primary antibodies overnight at 4°C. After washing for five times, the corneas were incubated with fluorescein-conjugated secondary antibodies overnight at 4°C. The flat mounts were examined under a ZEISS confocal laser scanning microscope (ZEISS, LSM880, Germany). Mouse anti-beta III Tubulin (1:150, ab78078, Abcam, United States) and chicken anti-Tyrosine Hydroxylase (1:150, ab76442, Abcam, United States) were used as primary antibodies. Alexa Fluor 488- and 594-conjugated secondary antibodies (Life Technologies, United States) were used.

### Corneal Sensitivity Assessment

Both preoperative and postoperative corneal sensation of rats undergoing capsulorhexis was measured by Aesthesiometer Cochet-Bonnet 12/100 (Luneau Technology, France) at the central cornea. Control and SCGx rats received capsulorhexis on their right eyes and had left eye corneal sensitivity measured 24 h after the right eye surgery. Corneal sensitivity was measured on awakened rats by two investigators together. Before the test, the rats were placed on the operating table for 10 min to accommodate with the surrounding environment. One investigator held the rat head and upper body with the left hand and held the rat tail with the right hand. The other investigator gently opened the upper and lower rat eyelids and applied the Cochet-Bonnet esthesiometry to the corneal center. The filament is applied perpendicular to the central portion of the cornea until there is a slight bend of the filament. The initial length is 6.0 cm (also the maximum length) and a 0.5-cm decrease is made each time until a consistent blink of the rat was observed. Each rat was measured three times and the average value was calculated as the threshold of corneal sensitivity. The assessment was repeated by two investigators.

### Electrophysiological Signature of Trigeminal Ganglion Neurons

Five male rats (weight 450–520 g) were used for baseline bioelectrical signal recording from the left trigeminal ganglia (TG). Twenty male SD rats were randomly divided into two groups. One group had right-sided SCGx surgery (SCGx_R) and had the lens extraction 14 days afterward, and the other group was set as the control group (Con_Post) having lens extraction only. The bioelectrical signals of left side trigeminal ganglion neurons were recorded by CerePlex Direct (Blackrock Microsystems, Salt Lake City, UT, United States) *in vivo* multi-channel neural signal acquisition system 1 day after the surgical stimuli. The signal data were analyzed by the software Neuroexplorer.

### Statistics

The paired *t*-test was used for the Visual Analog Scale (VAS) score comparison between patients receiving only one eye surgery and two eye surgeries. One-way ANOVA was used for the comparison of pain scores among the unilateral group and bilateral cataract surgery groups. The non-parametric Kruskal-Wallis test was used rabbit CSF3 gene expression at different time points after the first eye surgery. Pairwise comparisons were conducted between groups. The non-parametric Mann-Whitney *U*-test was used for rabbit CSF3 protein levels in the 1W group and SCGx_R group, for the fold change of rat Ly6G positive cells in the 1W group and SCGx_R group. An unpaired *t*-test was used for the fold change of rat Ly6G positive cells before and after fucoidan treatment. One-way ANOVA was used for the comparison of corneal sensitivities and for TG electrophysiological signatures among different groups, with multiple comparisons were conducted. The Chi-square test was used to compare categorical variables. The value *p* < 0.05 was defined as statistically significant.

## Results

### Patients Complained of More Pain After the Second Eye Cataract Surgery

**FIGURE 1:**
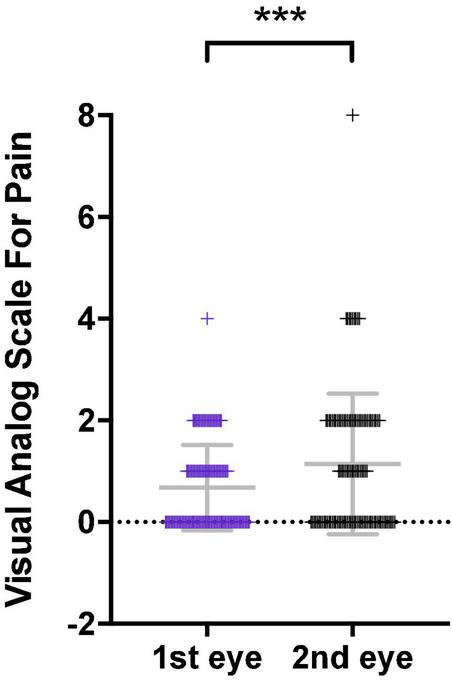
Pain visual analog score (VAS) comparison between patients receiving the first eye and the second eye cataract surgery. *n* = 112. The score is significantly higher in the second eye surgery group than the first eye surgery group. Significant difference: ***P < 0.001.

A total of 148 patients completed the pain score questionnaire. Both the VAS median and Wong-Baker Faces Pain Rating Scale reported pain score was 0 after the first cataract surgery. The score increased to 1 (*P* <0.001) and 2 (*P* = 0.009) after the second cataract surgery (Table 2). The mean VAS increased from 0.68 after the first eye surgery to 1.14 after the second eye surgery (*P* = 0.02) (Figure 1). There was no difference in VAS between the unilateral cataract group and the first-eye group (*P* = 0.7). In total, 62.5% of patients receiving the second eye surgery at a 1-week interval after their first eye surgery complained of more pain (Table 3), while the proportion dropped to 25% when the interval extended to 1 month (Table 3). The questionnaire results suggested the patient indeed perceived more pain during the second eye cataract surgery and such noxious feeling reached a peak when the interval between the two eye surgeries is 1 week.

**TABLE 2:** Pain score questionnaire analysis.

Demographic data	Unilateral cataract	Bilateral cataract		*P*
		First eye	Second eye	
Patients (n)	36	112		N/A
Mean age (y) ± SD	67.1 ± 8.3	66.9 ± 10.1		0.91
Gender (Male/Female)	11/25	50/62		0.14
OD/OS (n)	19/17	66/46	46/66	0.027
**Median (range)**				
VAS pain	0 (0,2)	0 (0,4)	1 (0,8)	<0.001
Wong-Baker Faces	0 (0,2)	0 (0,4)	2 (0,8)	0.009
**Mean ± SD**				
VAS pain	0.58 ± 0.76	0.68 ± 0.84	1.14 ± 1.37	<0.001
Wong-Baker Faces	0.83 ± 0.99	0.95 ± 1.03	1.32 ± 1.43	0.009

**TABLE 3:** Pain scores at different surgical intervals for bilateral cataract surgery.

	1W		2W		3W		≥1M	
	First eye	Second eye	First eye	Second eye	First eye	Second eye	First eye	Second eye
Patients (n)	32		34		18		28	
Mean age (y) ± SD	65.8 ± 9.4		67.0 ± 10.2		66.8 ± 12.1		68.2 ± 9.1	
Gender (Male/Female)	14/18		16/18		6/12		14/14	
More pain (%)	25.0	62.5	29.4	35.3	33.3	27.8	32.1	25.0
**Median (range)**								
VAS pain	0 (0,2)	2 (0,8)	0 (0,4)	1 (0,4)	1 (0,2)	1 (0,2)	1 (0,2)	0 (0,2)
Wong-Baker Faces	0 (0,2)	2 (0,8)	0 (0,4)	2 (0,4)	2 (0,2)	2 (0,2)	2 (0,2)	0 (0,2)
**Mean ± SD**								
VAS pain	0.63 ± 0.82	1.97 ± 1.72	0.71 ± 0.96	1.09 ± 1.27	0.67 ± 0.67	0.78 ± 0.79	0.57 ± 0.73	0.61 ± 0.73
Wong-Baker Faces	0.81 ± 0.98	2.13 ± 1.73	0.94 ± 1.11	1.18 ± 1.29	1.11 ± 0.99	1.11 ± 0.99	0.86 ± 0.99	0.86 ± 0.99

### First Eye Surgery-Induced Contralateral Trigeminal Responsiveness

**FIGURE 2:**
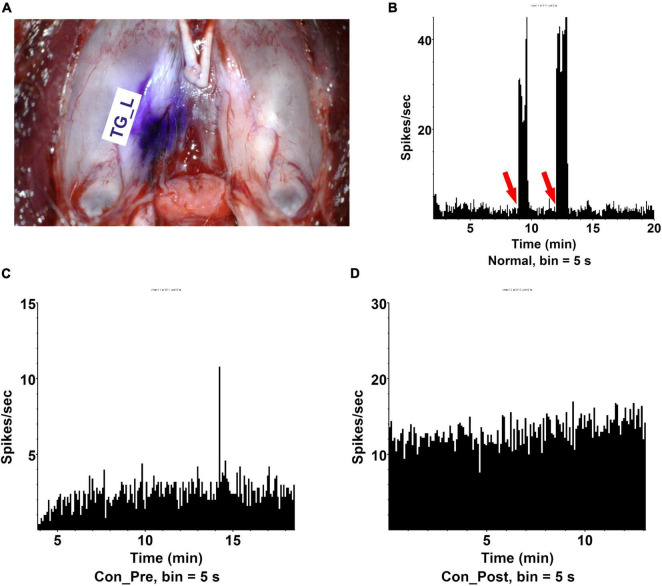
The first eye lens extraction induced more contralateral eye pain. **(A)** The dissection of trigeminal ganglia and the position of electrode collecting signals (TG_L). **(B)** Cornea stimulation could effectively induce trigeminal ophthalmic nerve branch firing (spike) recorded by electrophysiology. Red arrow: aesthesiometer touching the cornea. **(C)** Trigeminal ganglia (TG) spikes per second in a rat receiving no lens extraction (Con_Pre). The recording time was 30 min and signals were recorded every 5 s. *n* = 5. **(D)** TG spikes per second in a rat receiving OD lens extraction (Con _Post). The recording time was 30 min and signals were recorded every 5 s. *n* = 10.

Though more intense pain was widely reported during the second eye cataract surgery than the first eye surgery (Tan et al., 2011; Ursea et al., 2011; Adatia et al., 2015; Jiang et al., 2015; Yu et al., 2016; Liu et al., 2020), we examined this observation in the animal model. Capsulorhexis was conducted in the right eye of the rat and electrophysiological signatures were recorded in the left side TG. Nociceptors on the cornea had their cell bodies located in TG (Guerrero-Moreno et al., 2020), therefore, recording the pulse inside the contralateral TG of the surgical eye could directly reflect the nociceptive situation in the fellow eye. The trigeminal nerve originates from TG divides into three large branches: the ophthalmic (V1), maxillary (V2), and mandibular (V3), each of which innervates a distinct orofacial region. We placed the electrode in the TG close to the ophthalmic nerve (V1) (Supplementary Figure 1). Stereotactic staining indicated the electrode reached the TG V1 area (Figure 2A). Stimulation on the cornea by an aesthesiometer could successfully induce trigeminal nerve firing (spike) above the baseline (Figure 2B), which confirmed the location specificity of the electrophysiological recording. There is a dramatic increase of spike frequency in the left TG in the rats who received the first eye lens extraction (Con_Post, 10.6 spikes per second) compared with the rats free from surgery (Con_Pre, 1.74 spikes per second) (Figures 2C,D).

### Cytokine Activity Is the Top Functional Signature in the Contralateral Eye Aqueous Humor

**FIGURE 3:**
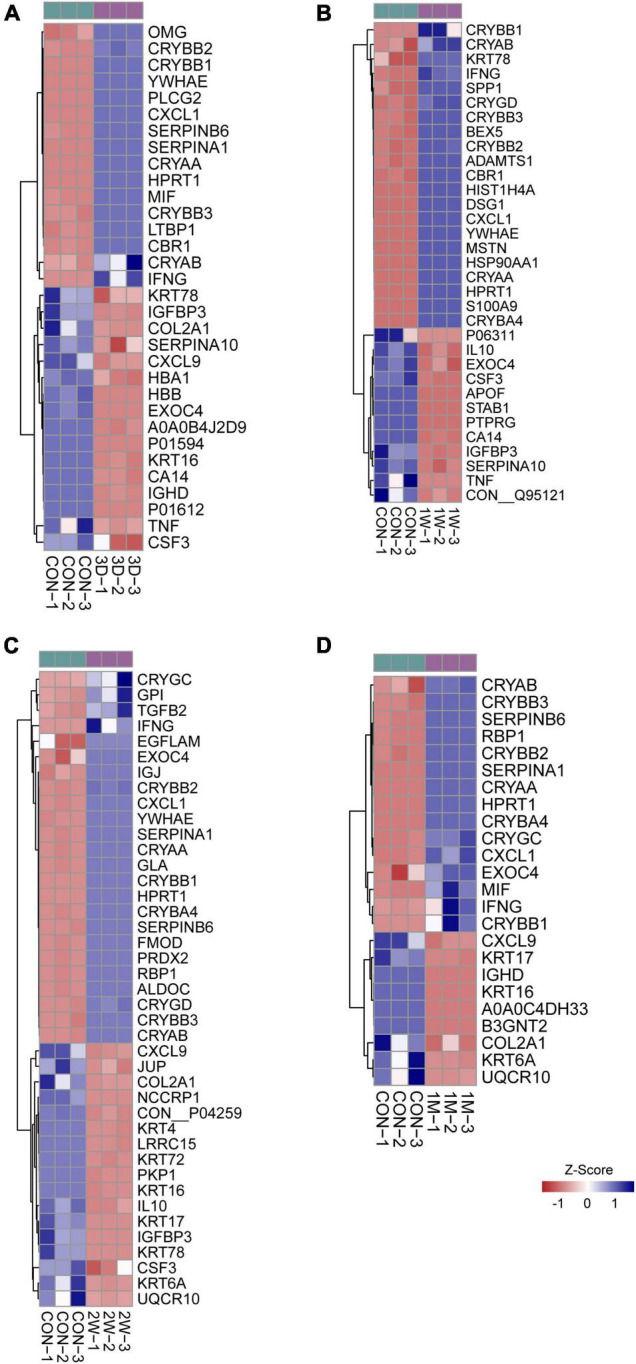
Heatmap of differentially expressed proteins in mass spectrometry. Comparison between the aqueous humor collected from control patients before receiving the first eye cataract surgery (Con) and the second eye of the patients having their first eye cataract surgery 3 days ago (3D) **(A)**, 1 week ago (1W) **(B)**, 2 weeks ago **(C)**, and 1 month ago **(D)**.

**FIGURE 4:**
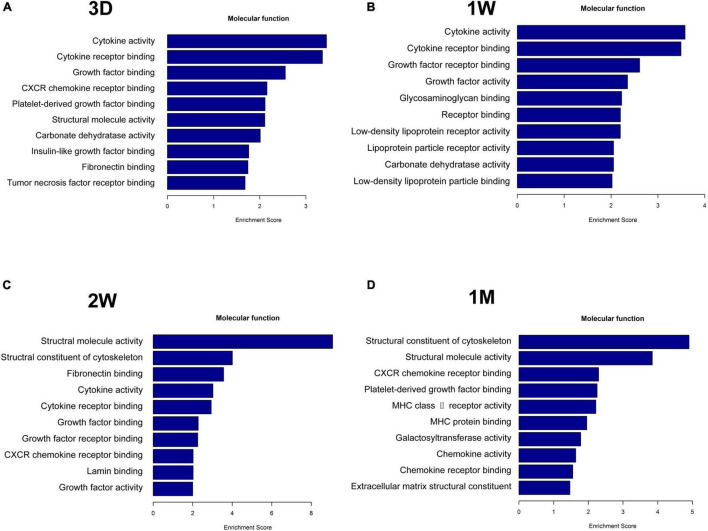
Functional analysis of differentially upregulated proteins in the fellow eye aqueous humor detected by mass spectrometry. The fellow eye aqueous humor was collected at **(A)** 3D, **(B)** 1W, **(C)** 2W, and **(D)** 1M post the first eye cataract surgery (PFC).

To uncover the mechanisms underlying the contralateral eye hypersensitivity, we conducted mass spectrometry with aqueous humor collected from the control (first eye) and the contralateral eye at different time points, post the first eye cataract surgery (PFC). A total of 32, 33, 41, and 25 differentially expressed proteins between the control and the contralateral eye were identified at 3D, 1W, 2W, and 1M PFC (Figure 3). The upregulated and downregulated protein numbers in the contralateral eye were shown in the volcano plot (Supplementary Figure 2). Molecular functions of the differentially expressed proteins were analyzed to decipher their unique functional signatures (Figure 4). To be specific, the top gene ontology (GO) terms include “cytokine activity,” “cytokine receptor binding,” and “growth factor activity,” especially in the 1W group. Cytokines have long been involved in the initiation and the persistence of pain by stimulating nociceptive sensory neurons, and more importantly, are associated with contralateral hyperalgesia or allodynia development (Zhang and An, 2007). In summary, mainly cytokine-mediated reaction is suggested contributing to the hyperalgesia in the contralateral eye PFC, with the reaction pattern various along time.

### First Eye Surgery Leads to the Increase of CSF3 in the Fellow Eye

**FIGURE 5:**
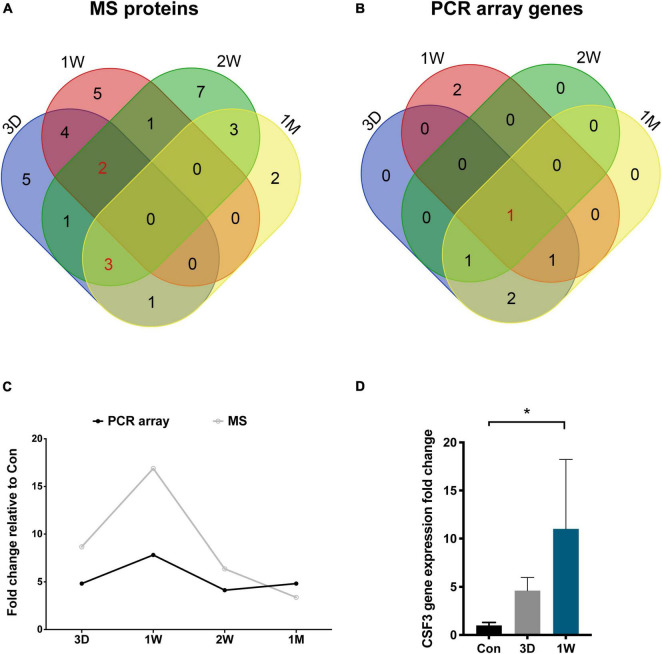
CSF3 is the key cytokine upregulated in the aqueous humor after contralateral eye cataract surgery. **(A)** Venn diagram for the mass spectrometry detected proteins in the aqueous humor collected at four time points. **(B)** Venn diagram for the PCR array detected genes in the aqueous humor collected at four time points. CSF3 is the only cytokine shared by all four time points. **(C)** Both the gene and protein expression of fellow eye CSF3 peaked at 1 week after the first eye cataract surgery. **(D)** Fellow eye CSF3 expression in the rabbit model with cataract surgery in their first eye. Significant upregulation of CSF3 was detected at 1 week PFC. Significant difference: *P < 0.05.

To reveal the key afferent protein in the second eye, we searched for the common protein shared by the four aqueous humor collecting times and found that no protein showed significant upregulation at all times (Figure 5A). Nevertheless, there are five proteins including structure constituent KRT16 and COL2A1, cytokine CXCL9 and CSF3, and IGFBP3 shared by three-time slots (Figure 3).

To confirm our observation in mass spectrometry, we applied a PCR array that can detect 90 cytokines. Surprisingly, CSF3 was significantly upregulated in the contralateral eye at all the timeslots tested, lasting from 3 days to 1 month PFC (Figure 5B and Table 4). To sum up, both the gene expression and the protein level of CSF3 dramatically increased in the second eye aqueous humor compared with the one-eye only cataract surgery aqueous humor. When plotted together, both CSF3 gene and protein expression levels peaked at 1 week (1W) PFC, with the fold change related to the first eye control to be 7.83 and 16.89, respectively (Figure 5C).

**TABLE 4:** Differentially up-regulated genes in the fellow eye surgery group relative to the control group detected by PCR array.

Group	Gene symbol	Fold change	*P* value
3D vs. Con	CSF3	4.83	0.038
	CXCL9	4.50	0.022
	CCL11	2.82	0.018
	MIF	2.34	0.018
	TNFα	2.22	0.041

1W vs. Con	CSF3	7.83	0.023
	MIF	3.06	0.043
	IL10	2.86	0.001
	CXCL2	2.06	0.027

2W vs. Con	CXCL9	11.66	0.023
	CSF3	4.13	0.028

1M vs. Con	CSF3	4.83	0.038
	CXCL9	4.50	0.022
	CCL11	2.82	0.018
	MIF	2.34	0.018
	TNFα	2.22	0.041

To avoid patient individual differences, we further constructed a rabbit model of lensectomy with phacoemulsification for the validation of the CSF3 expression pattern. Phacoemulsification was performed in the first eye of the rabbit, and aqueous humor was collected from the first eye before surgery as a control (Con). The second eye aqueous humor samples were collected at 3D, 1W, and 1M PFC. The qPCR for CSF3 was conducted with these samples. Consistent with the observations in humans, rabbit CSF3 in the fellow eye was remarkably higher than in the first eye, with the fold change being 4.61 and 11.01 at 3D and 1W. The CSF3 expression was back to normal level at 1M. Concordantly, the highest CSF3 level was detected at 1W (Figure 5D). In summary, in both patients and rabbit models, we identified an increase in CSF3 expression in the aqueous humor of the contralateral eye, especially 1 week after the first eye surgery.

### Contralateral Increase in CSF3 Is Related to Sympathetic Nerves

**FIGURE 6:**
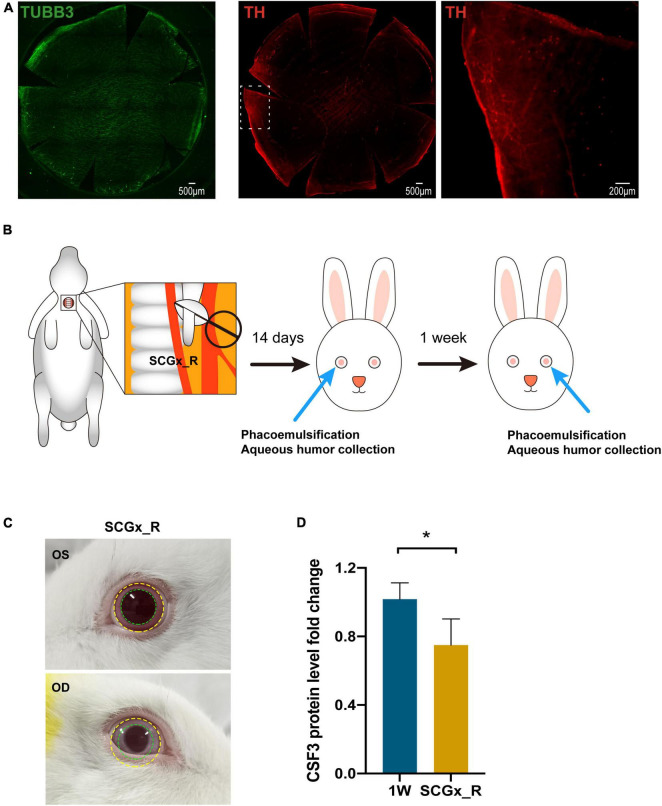
Regulation of contralateral CSF3 by the surgical side sympathetic nerves. **(A)** Immunostaining of sensory nerves and sympathetic nerves in rabbit cornea. Green: TUBB3; Red: TH. **(B)** Workflow of sequential surgeries conducted on the rabbit. **(C)** The ocular manifestations in rabbits with the right-side SCGx. Eye and pupil constriction were displayed. **(D)** The fellow eye aqueous humor CSF3 protein level was significantly decreased in the right-sided superior cervical ganglionectomy (SCGx) surgery SCGx_R rabbits when detected by ELISA. *n* = 5. Significant difference: *P < 0.05.

Rabbit cornea is abundant with nerve fibers when stained with neuronal-specific β-tubulin III (TUBB3) and sympathetic nerves marked by TH are mainly squeezed at the peripheral area, with the central cornea area occupied by the sensory neurons (Figure 6A). Preganglionic neurons originate from the spinal cord C8-T2 segments and synapse with postganglionic neurons in the superior cervical ganglion (SCG). The axons of these SCG postganglionic neurons further project into the eye and innervate the cornea (McDougal and Gamlin, 2015). To explore the relationship between sympathetic nerves and CSF3, we surgically removed the right SCG of the rabbit by superior cervical ganglionectomy (SCGx_R) (Figure 6B). Both the eye size (yellow circle) and the pupil size (green circle) on the right side reduced due to the SCGx (Figure 6C), indicating an ipsilateral control of the eye by the sympathetic nerves. According to our previous experience and the published studies, the sympathetic nerve terminal transmitter would release and activate the sympathetic activity in short term after SCGx (Martin et al., 1995). Therefore, we waited for 14 days till the paralytic phase after the rabbit had SCGx and conducted sequential phacoemulsification surgeries in the eyes afterward (Figure 6B). The aqueous humor CSF3 protein level of the SCGx_R rabbits and the first eye lensectomy with phacoemulsification only rabbits (1W) was measured with ELISA. The CSF3 protein level in the fellow eye declined by 25% with SCGx (Figure 6D, *P* = 0.029), suggesting the association between sympathetic nerve activity and intraocular cytokine level.

### Contralateral Neutrophil Concentration Is Related to Sympathetic Nerves

**FIGURE 7:**
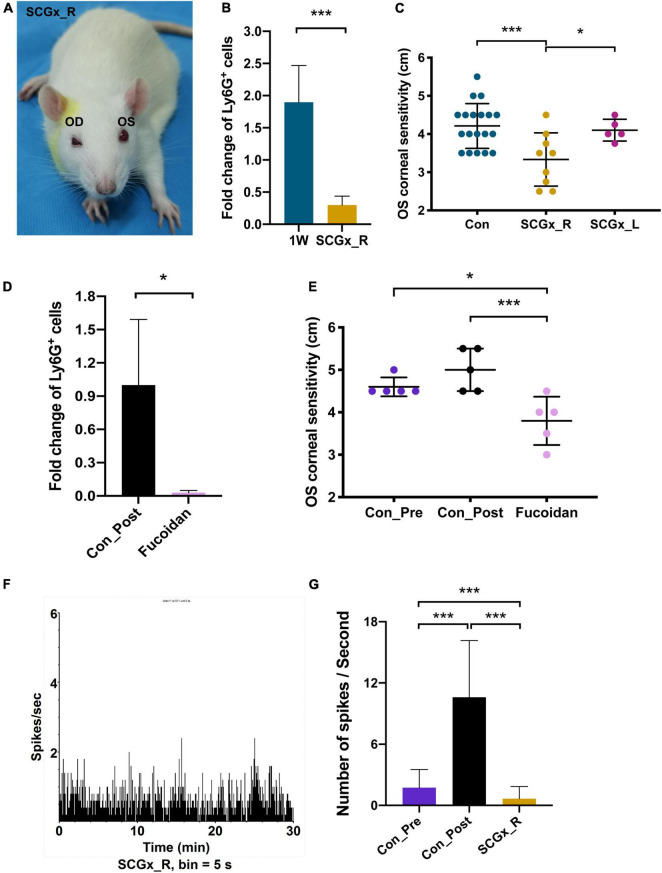
Regulation of fellow eye pain by the surgical side sympathetic nerves. **(A)** The ocular manifestations in the rat with the right-side SCGx. Blepharoptosis and pupil constriction were displayed. **(B)** Fold change of Ly6G^+^ cells in the left eye (OS) aqueous humor compared with the right eye (OD), in the control rats and the SCGx rats with capsulorhexis in their right eyes for 1 week. *n* = 20. **(C)** OS corneal sensitivity of the control rats (*n* = 19), rats with the right-side SCGx (*n* = 9) and rats with the left-side SCGx (*n* = 5). Rats in all three groups received OD capsulorhexis 1 week before corneal sensitivity was measured. **(D)** Intravenous application of fucoidan could effectively eliminate Ly6G^+^ cells in the aqueous humor. **(E)** OS corneal sensitivity decreased dramatically after capsulorhexis with fucoidan injection compared with the rats who had surgery only (Con_Post). *n* = 5. **(F)** TG spikes per second in a rat receiving OD lens extraction and the right-side SCGx. The recording time was 30 min and signals were recorded every 5 s. *n* = 10. **(G)** Quantifications of spike frequencies in different groups. An average of 1.74 spikes per second in the Con_Pre rats, 10.6 spikes per second in the Con_Post rats, 0.66 spikes per second in the SCGx_R rats. Significant difference: ***P < 0.001 and *P < 0.05.

Superior cervical ganglionectomy (SCGx) was also conducted in rats, and after blepharoptosis and pupil constriction displayed at the surgical site (Figure 7A), lens stimuli were applied. CSF3 (granulocyte colony-stimulating factor, G-CSF) is the granulocyte lineage development regulator. In line with the CSF3 ability that can stimulate the production and maturation of granulocytes, we observed an average of 1.9-fold more neutrophils in the rat second eye (OS) aqueous humor than the first eye (OD) aqueous humor 24 h after the corresponding eye surgery (Figure 7B). An example of neutrophil counting by flow cytometry is shown in the upper panel of Supplementary Figure 3. The percentage of Ly6G^+^ marked cells was 48.4% in the second eye and was 30.3% in the first eye. The disruption of sympathetic control of the ipsilateral eye resulted in a decline of the neutrophil content in the contralateral eye with 11.7% of Ly6G^+^ cells in OS compared with 28.5% in OD (Supplementary Figure 3). The fold-change of neutrophils in OS/OD was 0.3 with SCGx on the right side (SCGx_R), which is significantly lower than the normal rate (Figure 7B, *P* < 0.01).

### Disruption of the Surgical Side Sympathetic Network Mitigates the Contralateral Eye Pain

CSF3 as well as neutrophils have long been associated with inflammation and hypernociception (Fiset et al., 2003; Cunha et al., 2008; Schweizerhof et al., 2009; Lee et al., 2017). Therefore, the more abundant CSF3 and neutrophils suggest the hyperalgesia potential in the surgical contralateral eye. The cornea is abundant with sensory neurons. Immunostaining with TUBB3 shows that sensory nerve endings spread all over the rabbit cornea surface (Figure 6A). The great density of sensory nerves confers the cornea with high sensitivity. Thus, we measured corneal sensitivity as a behavior indicator for hyperalgesia after capsulorhexis. The Cochet-Bonnet aesthesiometer was used to evaluate the corneal sensation by perpendicularly touching the rat cornea with a nylon monofilament (Supplementary Figure 1). The perceived filament length was recorded as the sensitivity indicator. The larger the filament length, the more sensitive the cornea is. Consistent with the observations in flow cytometry, SCGx_R has the lowest fellow eye sensitivity among the three tested groups, with the filament reading being 3.3 cm (Figure 7C). The readings in the 1W group and the SCGx_L group are 4.2 and 4.1 cm, respectively, which are significantly higher than the SCGx_R group (*P* = 0.003) (Figure 7C), implying that sympathetic nerve disruption at the surgical side would efficiently mitigate the fellow eye pain.

To investigate whether the corneal sensitivity could reflect the neutrophil-induced inflammatory pain, we eliminated neutrophils and measured corneal sensitivity. Fucoidan is a class of sulfated polysaccharides that was reported to reduce neutrophil recruitment in a dose-dependent manner (Preobrazhenskaya et al., 1997). Fucoidan was applied by intravenous injection at 15 min after the right eye surgery and aqueous humor of the surgical eye was collected at 24 h post-surgery. Neutrophils in the aqueous humor decreased by 97% after the fucoidan injection (*P* = 0.047) (Figure 7D). Concordantly, the contralateral corneal sensitivity was dramatically relieved in the fucoidan-injected rats compared with the untreated rats (*P* = 0.004), but the difference with the pre- and post-surgery was indistinguishable due to the measuring range limitation of the aesthesiometer (Figure 7E). In summary, corneal sensitivity could faithfully reflect neutrophil-induced ocular pain and the post-surgery hyperalgesia is related to contralateral sympathetic nerves.

Finally, we applied TG electrophysiology to record neuronal pulse from the contralateral eye after SCGx. The spike frequency was quite low after SCGx (0.66 spikes per second) (Figure 7F), even lower than the wild-type rat receiving no lens extraction (Figure 7G, *P* < 0.001), suggesting milder nociception in the contralateral eye when the surgical side sympathetic network was disrupted.

## Discussion

**FIGURE 8:**
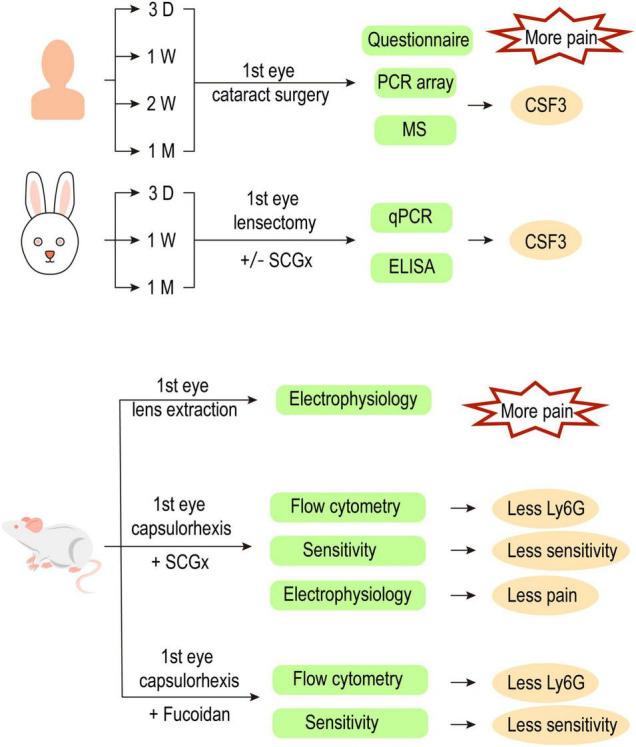
Workflow of this study.

In this study, we traced the cytokine profile alterations in the aqueous humor of the fellow eye after the first eye cataract surgery and identified CSF3 as a potential inducer of hyperexcitability in trigeminal corneal neurons. We further associated the sensory nerve activity with sympathetic nerve regulation by establishing rabbit and rat models (Figure 8). Our study suggests that CSF3 and sympathetic activity could serve as potential analgesic targets during ocular surgeries in the future.

Intraoperative comfort and postoperative inflammation are directly related to the degree of cooperation, satisfaction, and treatment effect of patients (Bardocci et al., 2011). In this study, we focused on the difference between intraoperative pain and the postoperative inflammation of bilateral eyes in patients with sequential cataract surgery. We found that the cytokine CSF3 was significantly higher in the contralateral eye compared with the first eye at all following time points, and subsequently, more neutrophils were recruited to the contralateral eye. CSF3 has been reported to sensitize nerves to mechanical stimuli in the skin, and the interruption of CSF3 signaling could reduce bone cancer pain (Schweizerhof et al., 2009). CSF3 and CSF3-driven neutrophil activity were reported to mediate ocular autoimmunity and could serve as a therapeutic target of uveoretinitis (Goldberg et al., 2016). Here we identified that CSF3 played a role in contralateral eye pain during bilateral cataract surgery, and this activity was associated with sympathetic nerves.

During the sequential phacoemulsification of bilateral cataract eyes, commonly, the doctor will first perform on the eye with worse visual acuity, heavier lens opacity, and higher lens nucleus hardness. Therefore, it is reasonable to conjecture that there would be more pain during the first operation, as more operative time and ultrasound energy would be required. However, in recent years, more and more observations indicated that there was more pain in the second eye compared with the first eye after the cataract surgery (Tan et al., 2011; Ursea et al., 2011; Adatia et al., 2015; Jiang et al., 2015; Yu et al., 2016; Liu et al., 2020). While in most of the previous studies the pain was measured by the VAS (Sharma et al., 2008; Gayadine-Harricham and Amzallag, 2017; Socea et al., 2020), individual differences in pain sensitivity and perception may lead to biases in the results of the study. Thus, in this study, we applied rabbit and rat lens surgery models and recapitulated CSF3 expression changes observed in patients. Questionnaire results from 112 randomly picked patients receiving bilateral cataract surgeries and 36 randomly picked patients receiving unilateral cataract surgery were analyzed and significant differences in pain perception between these two groups were identified. More interestingly, patients receiving the second cataract surgery 1 week post their first surgery complained of the most severe second eye pain, consistent with our CSF3 tendency, which implied our data could be validated in the real world.

In this study, we used both rabbits and rats as model animals to mimic the clinical manifestations observed in humans, but there are some limitations in the construction of these models. The rats we used for the electrophysiological experiment had ECLE, but we only performed capsulorhexis without lens extraction in rats used for the corneal sensitivity test and flow cytometry. The reason is that for the lens to be removed smoothly, we need to make an incision of about 150 degrees in the cornea. The suture of such a large corneal incision might have a potential influence on postoperative corneal sensitivity, and it would be difficult to collect the aqueous humor with such a large corneal incision. The aqueous humor of rats is around 20 μL. A microsyringe with a 33 G needle was used to collect aqueous humor 24 h after surgery. The large incision leads to a high failure rate of aqueous humor collection. If a 2.2 mm corneal incision is made and the incision is intermittently sutured with a 10–0 suture after capsulorhexis and hydrodissection without lens extraction, 10–15 μL of aqueous humor could be collected 24 h postoperatively. Therefore, we only performed capsulorhexis without lens extraction for rats used in the flow cytometry and corneal sensitivity tests. Nevertheless, both eyes underwent the same experimental procedures and the differences between the two eyes were compared.

The lens is free from sensory nerves. Consequently, the cataract surgical pain should be perceived by the sensory neuron endings in the surrounding tissues, such as the ciliary nerves and the corneal nerves. The possible surgical procedures that could generate pain are incisions on the cornea, gain of pressure in the anterior chamber, and iris touching during the performance. In addition, postoperative inflammation and psychological factors will contribute to nociception as well. Impulses originated from these ocular tissue sensory nerve endings gather at the trigeminal ganglion. The enriched CSF3 in the contralateral eye aqueous humor could directly interact with sensory nerve endings in the iris and ciliary body, to arouse nociception. In this study, we used corneal sensitivity as the behavior indicator for post-surgery nociception and recorded the electrophysiology of the trigeminal ganglion for pain sensitivity comparison. The electrophysiology difference is a direct evidence of the nociception difference between the two eyes.

At present, the behavioral texts for eye pain are rare. Corneal sensitivity measurement, as a relatively intuitive and convenient detection method, could reflect the threshold of corneal sensitivity to mechanical stimuli (Benítez-Del-Castillo et al., 2007). The cornea is the most sensitive tissue with abundant sensory nerve endings in the epithelium (Müller et al., 1997; Eisner, 2006). The cell body of the sensory neuron is located in the trigeminal nerve segment, and nerve fibers transmit *via* the ophthalmic division (V1) (Patel et al., 2009). Corneal neurons include three types of nociceptors: polymodal nociceptor, mechano-nociceptor, and cold-sensitive receptor (Belmonte et al., 2004). Corneal polymodal nociceptors and corneal mechano-nociceptors are likely to be involved in the perception of acute corneal pain caused by acute mechanical stimulation and respond to chemical stimulation, and inflammation pain (Armentia et al., 2000). The threshold of corneal mechano-nociceptor is significantly lower than that of the skin. Therefore, the corneal sensitivity measured could be related to nociception in TG. In our experiment, the corneal sensitivity test is to verify whether lens surgery will change the sensitivity threshold of the contralateral cornea to mechanical stimulation. Moreover, both corneal sensory neurons and uveal tissue sensory neurons are project through the ophthalmic division in TG. The TG electrophysiological signatures measured in our study is to ensure nociception signals collected by different sensory nerve endings were taken into account.

The filament length of the aesthesiometer is associated with the mechanical strength applied to the cornea. However, there are limitations in measuring the threshold. The Aesthesiometer Cochet-Bonnet is a device that contains a thin, retractable, nylon monofilament with a diameter of 0.12 mm. The length ranged from 60 to 5 mm of the nylon monofilament corresponds to pressures of 11–200 mg per 0.0113 mm^2^ (Martin and Safran, 1988). The length of the nylon filament was only 6 cm long, and in the length range of 4.5–6.0 cm, the corresponding pressures difference was small, and could not be distinguished very well. On the contrary, the corneal sensation measurement works very well in the middle of the measuring range, which is mainly used to demonstrate the role of SCGx. The reading difference of the pre- and post-surgery sensation is not as significant as we have expected, which is likely being influenced by the range of the measuring instruments. Anyhow, we have observed a significant difference in the electrophysiological results before and after surgery, so we don’t think the limitation of the corneal sensitivity measurement would influence our core conclusions generated from this study.

Bilateral eye inflammation is widely observed when only one eye get an actual injury. Unilateral ultraviolet radiation type B exposure-induced cataracts will lead to the sympathetic inflammatory reaction in the non-exposed fellow eye (Meyer et al., 2013). One eye injury causing bilateral uvea inflammation is the characterization of sympathetic ophthalmia (Damico et al., 2005). Such reaction being neuronal origin or aqueous humoral origin is still not clear. We applied SCGx to rabbits and rats, conducted phacoemulsification or simulated lens surgeries in one eye, and observed the decrease in the aqueous humor CSF3 protein level, neutrophil number, corneal sensitivity of the contralateral eye, indicating a direct association of SCGx with the fellow eye inflammation. Therefore, we provided evidence of sympathetic nerves mediating the intraocular inflammation in the fellow eye after the first eye lens surgery. We also demonstrated that the disruption of the surgical eye side sympathetic pathway, rather than the contralateral sympathetic pathway, is effective in reducing inflammation in the contralateral eye, suggesting an ipsilateral control mode of sympathetic nerves. However, how the sympathetic neuronal signals control CSF3 concentration in the fellow eye, and what would be the intermediate efferent factors, are still unknown. The association analysis between CSF3 and pain scores in the real world has not been studied yet. CSF3 is the top cytokine identified from the PCR array and the mass spectrometry in our study, and CSF3 directly regulates the neutrophil population, and we focused on neutrophils in this study. But there are other cell types that exist in the aqueous humor after the cataract surgery, as the Ly6G positive cells are the major but not the only cell type in the flow cytometry. The other cell populations are also very important for our understanding of the environmental change in the aqueous humor after cataract surgery. More exploration needs to be conducted in the future to address these gaps.

## Data Availability Statement

The datasets presented in this study can be found in online repositories. The names of the repository/repositories and accession number(s) can be found below: https://ngdc.cncb.ac.cn/omix/methods, OMIX690.

## Ethics Statement

The studies involving human participants were reviewed and approved by the Ethics Committee of Qingdao Eye Hospital. The patients/participants provided their written informed consent to participate in this study. The animal study was reviewed and approved by Animal Investigation Committee of Shandong Eye Institute.

## Author Contributions

BNZ and YH concepted the design. ZF, CF, BQ, XQ, and XL conducted the experiments. ZF, BZ, and BNZ did the statistical analysis. ZF and BNZ contributed to data interpretation and drafting of the manuscript. WL, BNZ, and YH did critical editing of the article. All authors contributed to the article and approved the submitted version.

## Conflict of Interest

The authors declare that the research was conducted in the absence of any commercial or financial relationships that could be construed as a potential conflict of interest.

## Publisher’s Note

All claims expressed in this article are solely those of the authors and do not necessarily represent those of their affiliated organizations, or those of the publisher, the editors and the reviewers. Any product that may be evaluated in this article, or claim that may be made by its manufacturer, is not guaranteed or endorsed by the publisher.

## Footnotes

1
https://ngdc.cncb.ac.cn/omix/methods
