# Supplementary material for: Sympathetic Nerve-Mediated Fellow Eye Pain During Sequential Cataract Surgery by Regulating Granulocyte Colony Stimulating Factor CSF3
Source: Front Cell Neurosci. 2022 Feb 24;16:841733. doi: 10.3389/fncel.2022.841733 (PMC8907920; doi:10.3389/fncel.2022.841733)
Supplement: Supplementary file 1 [file Data_sheet_1.pdf]

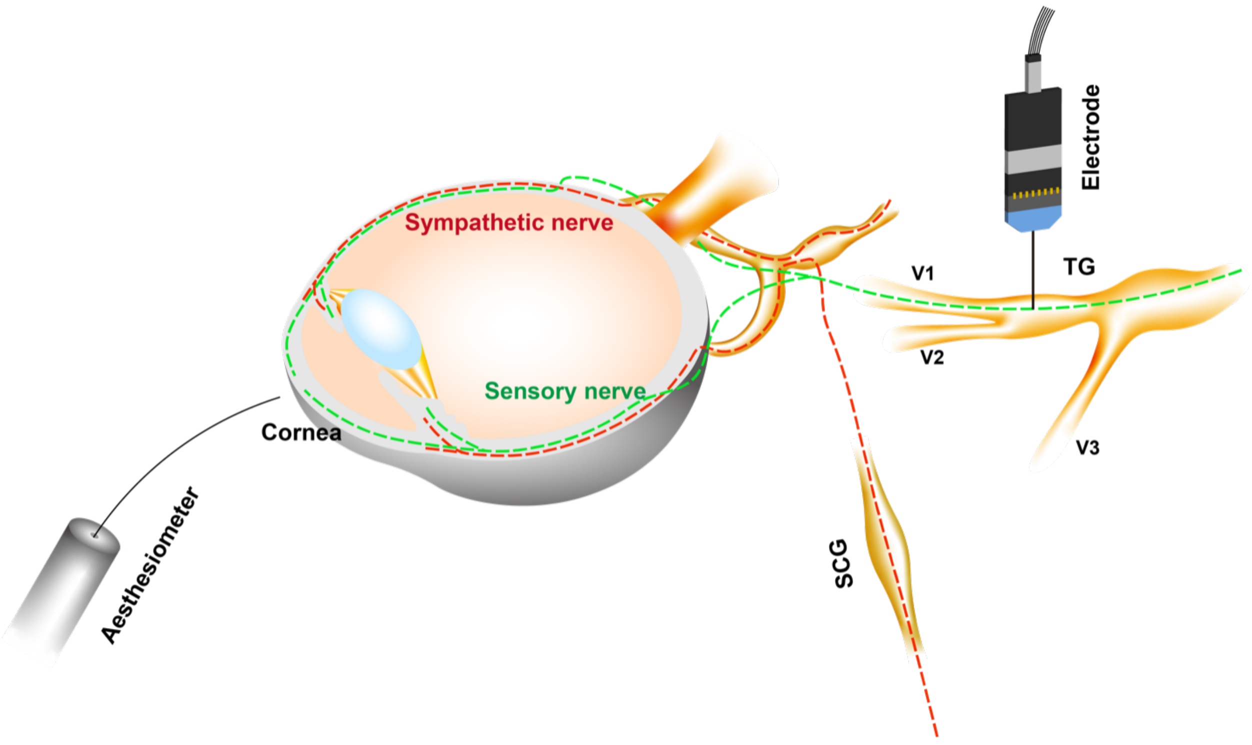

Supplementary Figure 1. Schematic diagram of ocular nerves and the behavior and electrophysiological tests of these nerve activities.

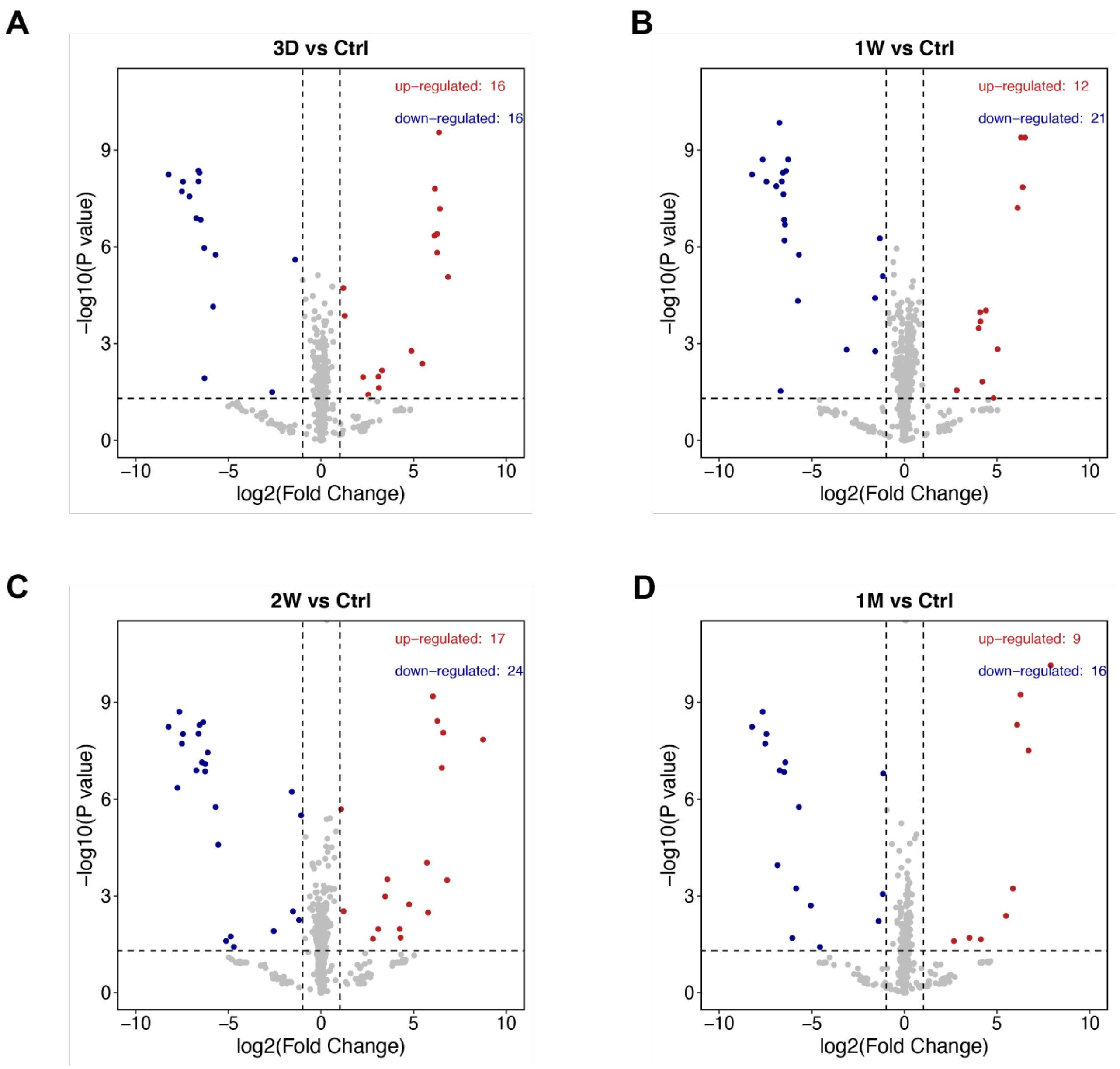

Supplementary Figure 2. Volcano plots of differentially expressed proteins in mass spectrometry.

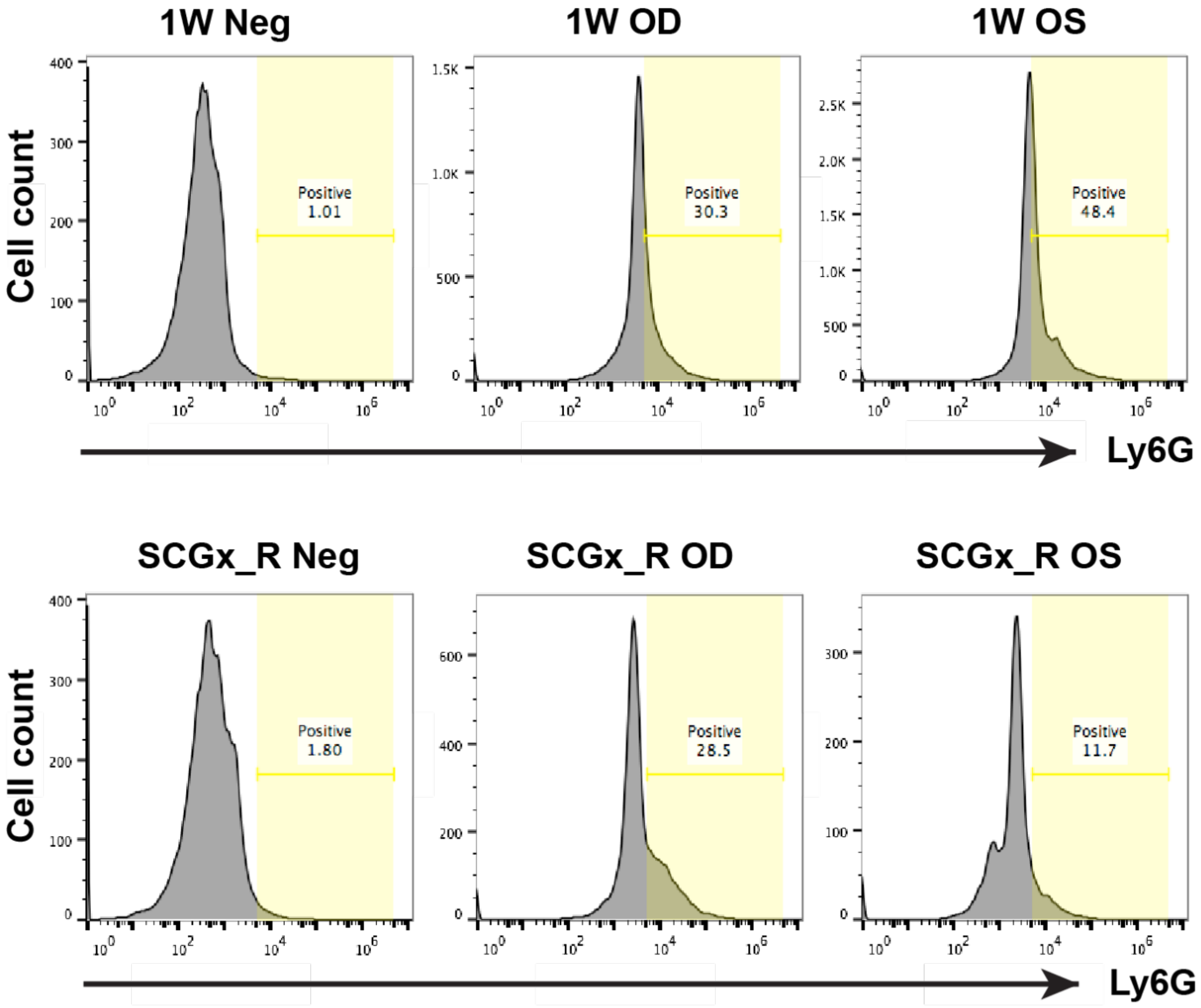

Supplementary Figure 3. Examples of flow cytometry results of aqueous humor samples collected from a control rat and a SCGx rat, respectively.
